# Maternal positive coparenting and adolescent peer attachment: Chain intermediary role of parental involvement and parent–child attachment

**DOI:** 10.3389/fpsyg.2022.976982

**Published:** 2022-10-10

**Authors:** Wanghua Ji, Yang Yang, Ying Han, Xiaohua Bian, Yunhong Zhang, Junqi Liu

**Affiliations:** ^1^School of Management, Henan University of Traditional Chinese Medicine, Zhengzhou, China; ^2^School of Educational Science, Henan Finance University, Zhengzhou, China; ^3^School of Educational Science, Zhengzhou Normal University, Zhengzhou, China

**Keywords:** maternal positive coparenting, parental involvement, parent–child attachment, peer attachment, adolescents

## Abstract

This study investigated the relationship between maternal positive coparenting and adolescent peer attachment, and the intermediary role of parental involvement and parent–child attachment in 1,807 families using the maternal positive coparenting scale, the parental involvement scale, and the parent and peer attachment scale. The results showed that maternal positive coparenting behaviour, parental involvement, parent–child attachment, and peer attachment had significant positive relationships, and maternal positive coparenting had a positive correlation with adolescent peer attachment. Moreover, parental involvement and parent–child attachment played a significant mediating role between maternal positive coparenting behavior, including unity and consistent behavior, and adolescent peer attachment, respectively, which consisted of a sole intermediary role of parental involvement; a single intermediary role of parent–children attachment; and a chain intermediary role of parental involvement and parent–children attachment. Hence, maternal positive coparenting was positively associated with adolescent peer attachment, in which parental involvement and parent-child attachment served as a crucial bridge.

## Introduction

Peer attachment is the stable and lasting affective associations among peers, which includes trust, reliance, and sharing personal thoughts and emotions (Bowlby, 1979; Armsden and Greenberg, 1987; Xin et al., 2014). Adolescence is a critical period for developing individual peer attachment, which has an important impact on adolescents’ mental health and social adaptation (Armsden and Greenberg, 1987; Laible et al., 2000). Developing peer attachment affects inner traits and emotions, such as self-esteem, loneliness, depression (Liu et al., 2020), low mood (Lee and Park, 2017), academic self-efficacy and achievement (Llorca et al., 2017), and metal resilience (Oldfield et al., 2018), and predicts external problems and behaviours, such as internet addiction (Yang et al., 2016; Chen et al., 2018), bullying behaviours (Murphy et al., 2017), prosocial behaviour (Oldfield et al., 2016; Schoeps et al., 2020), and so on. Peer attachment is based on early bonding experiences with the parents during the transition to adolescence (Oldfield et al., 2016), which is affected by many family factors, especially interactions between children and parents (Meeus et al., 2002; Allen, 2008; Xiaoyan et al., 2011). Previous studies have examined the role of family influences in peer attachment. However, these studies did not focus on positive family factors. Therefore, it has great theoretical and practical significance to investigate positive family factors and their underlying mechanisms.

Recently, researchers have paid more attention to family systems theory, which divides family interactions as a whole into a marital binary system, a father-child, mother–child binary system, and a parent–child ternary system (Belsky et al., 1995), involving multiple levels of influence within families, such as interaction patterns with different caregivers (Cox and Paley, 1997). Beyond the father-child and mother–child system, family research is now focused on the more diversified ternary system and the interaction between different systems (Minuchin, 1985; Doherty and Beaton, 2004). The spillover hypothesis holds that emotions or behaviors generated in one subsystem (e.g., the parent–child ternary system) are similarly expressed in another (e.g., the parent–child subsystem; Erel and Burman, 1995). For example, the mother’s positive coparenting may be reflected in the maternal involvement. The crossover hypothesis states that the emotions or behaviors of one of the interacting parties in a certain subsystem of the family (e.g., mother’s positive coparenting) will affect those of other parties in other subsystems (e.g., paternal parenting involvement) (Bolger et al., 1989). In which coparenting is more important. Coparenting is a ternary relationship system composed of parents and children, which refers to an alliance formed by parents or caregivers in the process of child rearing, and includes the positive or negative attitudes of one parent towards the other (Feinberg and Hetherington, 2007; Chang and Xinchun, 2015). Positive coparenting and negative coparenting have different effects on family function, adolescent development, and adaptation. Positive coparenting behaviour occurs when one family member responds positively to the other’s parenting behaviour and goals, while negative coparenting occurs when one party reacts negatively to the other’s parenting behaviour and goals (McHale, 1997). Although coparenting behaviour has been examined in previous family studies, these studies focused on negative coparenting behaviour and the adverse effects of overall collaborative parenting behaviour on adolescents’ psychological development (Zou and Wu, 2019; Riina et al., 2020). Although recent research has focused more on positive psychology perspectives, such as the influence of positive behaviour on human health development (Xi et al., 2019), the underlying mechanisms of how positive coparenting was associated with on adolescent psychological development are little known (Leary and Katz, 2004). Therefore, exploring the association of positive collaborative parenting behaviour with adolescents’ peer attachment may promote both parental awareness practice of positive collaborative parenting behaviour and healthy adolescent peer attachment development, thereby playing an important role in developing more targeted practical interventions to improve the quality of family education (Goede et al., 2009; Lin et al., 2014).

As the main family caregiver, mothers have more frequent interactions with adolescents than fathers, and mothers’ positive cooperative parenting behaviours have a larger influence on both adolescents and the entire family than fathers’ (Barth et al., 2020). Therefore, this study mainly focused on mothers’ positive collaborative parenting. Guided by family systems theory, mothers’ positive cooperative parenting behaviour is a ternary subsystem of family interaction that affects the father-child and mother–child systems and whole family atmosphere. When mothers practice more positive collaborative parenting behaviour, the whole family forms a warm atmosphere, which promotes positive interactions among teenagers, promoting positive peer attachment development (Allen et al., 1996). Also, when mothers’ and fathers’ childrearing behaviours are consistent when interacting with teenagers, the teens acquire a belief that parents support and understand each other and are reliable. In turn, teens apply this belief to interpersonal communication with their peers, which promotes healthy peer attachments (Tian et al., 2014). Previous studies have shown that mothers’ collaborative parenting has an important impact on adolescent peer attachment (Brown et al., 2010). Therefore, this study proposed Hypothesis 1: mothers’ positive cooperative parenting behaviour was positively associated with adolescents’ peer attachment.

### Mediating effect of parenting involvement

Parental involvement is an important way for parents to interact with their children, and refers to parents’ involvement in behavioral, emotional and cognitive aspects in the process of raising children (Lamb, 2004). According to the family systems theory spill over effect, emotional experiences formed in one family subsystem (or one aspect of a family subsystem) affect the emotional experiences of other family subsystems (or other aspects of a family subsystem; Erel and Burman, 1995). For example, mothers’ positive collaborative parenting behaviour in the parent–child ternary system spills over and affects mothers’ parenting behaviour in the binary system (Martin et al., 2017). Dykas and Cassidy (2011) found that fathers’ parenting involvement behaviour was significantly affected by mothers’ positive cooperative parenting behaviour.

Since parents are the main caregivers and psychological supporters of adolescents, their parenting input is an important source of adolescents’ sense of security and trust (Main et al., 1985; Becker-Stoll et al., 2008). Previous studies have also shown that trust and security are important factors for predicting peer attachment development (Dykas and Cassidy, 2011). In their interactions with peers, adolescents with a sufficient sense of trust and security engage more positive interaction modes with others. Therefore, the quality of peer attachment has an important relationship with parents’ ability to provide the parenting input their children need (Jones and Cassidy, 2014; Tu et al., 2014). Hypothesis 2 proposes that parenting involvement plays a mediating role between mothers’ positive cooperative parenting behaviour and adolescents’ peer attachment.

### Mediating role of parent–child attachment

Parent–child attachment refers to the deep, stable and lasting emotional bond between parents and children. The parent–child system is clearly influenced by the parent subsystem—the parent–child system belongs to the parent subsystem, and parent–child attachment belongs to the parent–child system (Armsden and Greenberg, 1987). Spill over and crossover effects in Family systems theory assert that mothers’ active cooperative parenting may affect both father-child attachment and mother–child attachment (Minuchin, 1985; Martin et al., 2017). When mothers and fathers are united in their parenting behaviours, the family atmosphere is warmer and more harmonious. In this atmosphere, teenagers have a more positive emotional experience regarding their parents, and are more likely to form healthy parent–child attachments. Previous studies have shown that active collaborative parenting has an important impact on the development of adolescent parent–child attachment (Zou et al., 2020). As individuals enter adolescence, they establish a more complete attachment experience psychological state (Main et al., 1985). Developing peer attachments is an important psychological task for adolescents. According to the internal working model of attachment, an individual will form a stable internal mechanism for responding to the outside world through interacting with parents in early stages. This mechanism will become the basis of interaction between individuals and others in the future and affect how individuals react to others (Simpson et al., 1992). Therefore, the internal working model established during parent–child attachment will be applied to the peer interactions, affecting adolescent peer attachment (Ma and Huebner, 2008). Previous studies have shown that the development of adolescent peer attachment is significantly affected by parent–child attachment (Wu and Wang, 2014; Murphy et al., 2017). Therefore, this study proposed Hypothesis 3: parent–child attachment plays a mediating role between mothers’ active collaborative education and adolescent peer attachment.

### Chain mediating effect of parenting involvement and parent–child attachment

According to the family system theory spill over hypothesis, the emotion and behaviour of one individual or party (or in one aspect) will spill over and affect the emotional experience and behaviour of an individual and another party (or another aspect) in the process of family interaction (Martin et al., 2017). Parenting involvement belongs to the dual parent–child system. Paternal attachment and maternal attachment reflect a bi-directional relationship between parents and children, which differ from the family function of parenting involvement, but is also a subsystem of the binary parent–child system. Therefore, although both parenting involvement and parent–child attachment belong to the family binary subsystem, parenting involvement is a one-way parent–child system from top to bottom, whereas parent–child attachment is a two-way emotional connection reaction system (Cassidy, 2008). According to the spill over hypothesis, mothers’ emotional experience and behaviour formed by parenting will spill over to the mother–child attachment system to influence maternal attachment; and fathers’ emotional experience and behaviour formed by parenting will spill over to the father-child attachment system to influence paternal attachment. Previous studies have shown that mother–child attachment is significantly affected by mothers’ parenting involvement, and father-child attachment is significantly affected by fathers’ parenting involvement (Hou et al., 2018). According to the internal working model of attachment, parent–child attachment affects peer attachment development. Considering the family system theory’s crossover and spill over effects, mothers’ positive coparenting behaviour affects parents’ involvement in parenting, which affects parent–child attachment. Based on this, we proposed Hypothesis 4: parenting involvement and parent–child attachment are related to mothers’ positive cooperative parenting behaviour and peer attachment and play a chain intermediary role.

## Materials and methods

### Setting and participants

A total of 1901 traditional two-parent families, including parents and adolescents, participated in the study, with 1,807 providing valid data, for an effective response rate of 95.06%. Adolescents’ average age was 14.78 ± 1.90 years, with 928 boys (47.7%) and 879 girls (52.3%). Sixty-seven adolescents were only-children (3.7%) and 1,740 (96.3%) came from families with more than one child. The average subjective social economic status score (SSS) for the school and province were 5.28(SD = 1.75) and 5.41 (SD = 1.71; full range = 10), respectively. Fathers’ average age was 45.62 (SD = 12.40) years and mothers’ was 43.89 (SD = 6.15) years.

### Procedure

Parents and adolescents were administered different questionnaires. Adolescents completed their questionnaires in school, and took the parent questionnaires home for their parents to complete, and then returned them to the school for unified collection. The returned questionnaires were screened and those with missing pages or missing responses for more than three items were excluded. The data were recorded into IBM SPSS Statistics, version 22.0 and analysed using descriptive statistics, correlation analysis, difference analysis, bootstrap tests, etc.

### Measures

#### Mothers-reported mothers’ positive collaborative parenting questionnaire

This questionnaire was compiled by McHale (1997) and revised in China by Chang et al. (2017). There are 29 questions in the questionnaire, including four dimensions: unity, consistency, conflict, and demeaning, only two dimensions (integrity, consistency) of positive co-parenting were selected in this study. The higher the scores, the more collaborative parenting behaviours. In the evaluation version of the parent collaborative parenting questionnaire, the adolescent language style was modified to reflect mothers’ self-evaluation language. For example, the adolescent self-evaluation was changed from “when my father is restraining my behaviour, my mother supports his decision” to “when my husband is restraining my child’s behaviour, I support his decision.” The participants responded to the items on a 7-point Likert scale ranging from 1 (never) to 7 (always); higher scores indicate higher coparenting behavior. Confirmatory factor analysis indicated that this scale has good validity, with factor loading greater than 0.6(RMSEA = 0.061,CFI = 0.92,TLI = 0.92, SRMR = 0.047). The Cronbach’s α coefficients of the two dimensions were 0.91 and 0.95, respectively.

#### Parent-reported parenting involvement

The parenting involvement questionnaire developed by Wu et al. (2015) has good reliability and validity. Fathers and mothers have the same parenting input structure, measured using the same structure questionnaire (Fagan et al., 2014). This questionnaire has been widely used to measure parenting involvement (Wu et al., 2018) and is divided into three dimensions: interactivity, which measures the interaction between parents and children; accessibility, which measures how much parents connect with their child’s life; and responsibility, which measures the extent that parents are responsible for their children. The questionnaire has 56 items in the three dimensions, with scores on a five point scale from 0 to 4. Higher scores indicate higher levels of parenting involvement behaviour. In this study, the alpha coefficients for the three dimensions of parenting investment ranged from 0.94 to 0.97.

#### Adolescent-reported parent–child and peer attachment

This questionnaire was compiled by Armsden and Greenberg (1987) and translated and revised by Xiao and Chen (2009). It was completed by the adolescents and included three dimensions of parent and peer attachment: trust (e.g., understanding and respect), communication (e.g., communicating style), and alienation (e.g., anger and neglect). The questionnaire’s 25 items were scored on a five-point scale (1 = strongly disagree, 5 = strongly agree). Higher scores of trust and communication and lower scores of alienation indicated higher levels of attachment qualities. The reported Cronbach’s alphas of the three subscales were 0.80, 0.70, and 0.82, respectively.

### Data analysis

Data were analysed using IBM SPSS version 22. We analysed control and inspection of common method deviation. Reverse scoring and different subjects were used to eliminate the influence of common method bias. The single factor test method recommended was used to test the common method deviation in the collected data and showed that the first factor explained 30.55% variance (e.g., less than 40% of the standard). Therefore, common method bias was not serious in this study. Then descriptive statistic and Chain Mediated Effect were conducted.

## Results

### Descriptive statistics and correlations

Presents the means and standard deviations of all study variables and the results of the correlation analyses. The correlation matrix indicated that adolescent age was positively related to maternal adult anxiety. Meanwhile, adolescent age was negatively related to paternal adult anxiety and paternal harsh parenting. Family SES was not related to other variables. Both paternal and maternal avoidance and anxiety were positively and significantly related to harsh parenting, that is, the higher the father’s and mother’s avoidance and anxiety, the harsher their parenting styles. Both father’s and mothers’ levels of avoidance and anxiety were negatively and significantly related to parent–adolescent attachments, indicating that the higher the levels, the worse the parent–adolescent attachment development. Harsh parenting by both fathers and mothers was negatively and significantly correlated with parent–adolescent attachments, indicating that the harsher the parenting style, the worse the parent–adolescent attachment development.

Descriptive analyses were conducted to examine the means and standard deviations and the relations between the variables of maternal coparenting integrity, maternal coparenting consistency, father involvement, mother involvement, paternal attachment, maternal attachment, and peer attachment. Table 1 showed both maternal coparenting integrity and maternal coparenting consistency were positively and significantly related to peer attachment, which means the more positive behaviors of mothers, the higher level of peer attachment of children. And both mother’s coparenting integrity and consistency and parental involvement were also significantly positively correlated. Father and mother involvement and peer attachment show positive relationship significantly still, that is, the more parental involvement, the higher level peer attachment of children. Moreover, the results indicated that the correlation between other variables is also significant.

**Table 1:** Pearson correlations and descriptive statistics of the main study variables (*N* = 1807).

	1	2	3	4	5	6	7
1. Mother Coparenting Integrity	1						
2. Mother Coparenting Consistency	0.71^**^	1					
3. Father Involvement	0.50^**^	0.49^**^	1				
4. Mother Involvement	0.63^**^	0.62^**^	0.73^**^	1			
5. Paternal Attachment	0.31^**^	0.27^**^	0.36^**^	0.33^**^	1		
6. Maternal Attachment	0.23^**^	0.21^**^	0.10^**^	0.13^**^	0.28^**^	1	
7. Peer Attachment	0.30^**^	0.29^**^	0.29^**^	0.27^**^	0.34^**^	0.63^**^	1
*M*	29.26	46.13	3.24	3.39	81.14	73.64	77.56
SD	8.54	12.24	0.81	0.79	14.09	14.90	12.96

** *p* < 0.01.

### The chain mediated effect of parental involvement and parent–child attachment

**Figure 1:**
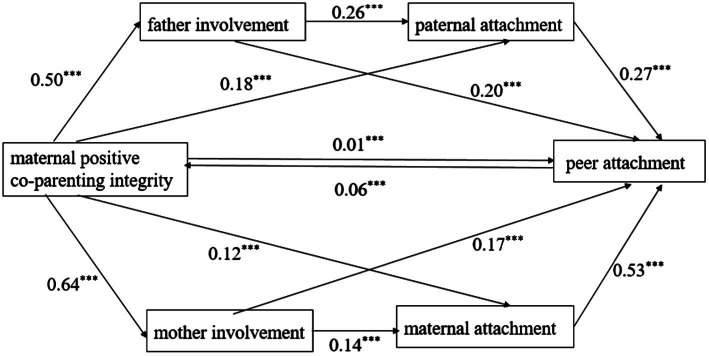
Chain mediating of parenting involvement and parent-child attachment between maternal positive co-parenting integrity and peer attachment. ***p < 0.001.

**Figure 2:**
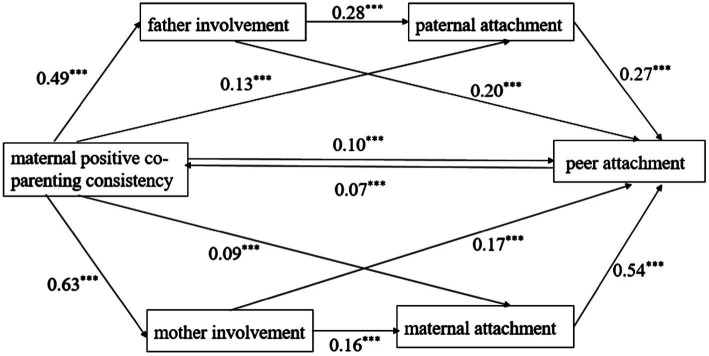
Chain mediating of parenting involvement and parent-child attachment between maternal positive co-parenting consistency and peer attachment. ***p < 0.001.

The SPSS macro process program compiled was used to produce 1,000 extractions. After controlling for gender, age, single child status, and subjective socio-economic status, we analysed the mediating effects of parent involvement and parent–child attachment on the relationships of mothers’ positive cooperative parenting integrity and consistency with adolescent peer attachment. The results are shown in Table 2, and the chain mediated effect is shown in Figures 1, 2.

**Table 2:** Analysis of chain mediated effect (*N* = 1807).

Indirect effect	*B*	*R* ^2^	95% CI
1 → 3 → 7	0.17	39%	[0.12, 0.23]
1 → 3 → 5 → 7	0.07	17%	[0.04, 0.10]
1 → 5 → 7	0.10	22%	[0.05, 0.14]
Total indirect effect	0.34	78%	[0.27, 0.41]
1 → 4 → 7	0.15	35%	[0.11, 0.20]
1 → 4 → 6 → 7	0.06	14%	[0.04, 0.07]
1 → 6 → 7	0.07	16%	[0.04, 0.09]
Total indirect effect	0.28	65%	[0.23, 0.34]
2 → 3 → 7	0.11	39%	[0.07, 0.16]
2 → 3 → 5 → 7	0.06	20%	[0.04, 0.08]
2 → 6 → 7	0.05	17%	[0.02, 0.08]
Total indirect effect	0.22	75%	[0.18, 0.28]
2 → 4 → 7	0.10	35%	[0.08, 0.13]
2 → 4 → 6 → 7	0.05	17%	[0.03, 0.05]
2 → 6 → 7	0.04	14%	[0.02, 0.06]
Total indirect effect	0.18	62%	[0.15, 0.22]

1, maternal positive coparenting integrity; 2, maternal positive coparenting consistency; 3, mother involvement; 4, father involvement; 5, maternal attachment; 6, paternal attachment; 7, peer attachment.

The results indicate that mothers’ parenting involvement and mother–child attachment have a significant chain mediating effect on the relationship between maternal positive coparenting integrity and adolescent peer attachment (95% CI: 0.04, 0.10). Maternal positive coparenting integrity was indirectly related to adolescent peer attachment through mothers’ parenting involvement (95% CI: 0.12, 0.23). Maternal positive coparenting integrity was indirectly related to adolescent peer attachment through mother–child attachment (95% CI: 0.05, 0.14).

The results also implicate that fathers’ parenting involvement and father-child attachment have a significant chain mediating effect on the relationship between maternal positive coparenting integrity and adolescent peer attachment (95% CI: 0.04, 0.07). Maternal positive coparenting integrity was indirectly related to adolescent peer attachment through fathers’ parenting involvement (95% CI: 0.11, 0.20). Maternal positive coparenting integrity was indirectly related to adolescent peer attachment through father-child attachment (95% CI: 0.04, 0.09).

The results indicate that mothers’ parenting involvement and mother–child attachment have a significant chain mediating effect on the relationship between maternal positive coparenting consistency and adolescent peer attachment (95% CI: 0.04, 0.07). Maternal positive coparenting consistency was indirectly related to adolescent peer attachment through mothers’ parenting involvement (95% CI: 0.07, 0.16). Maternal positive coparenting consistency was indirectly related to adolescent peer attachment through mother–child attachment (95% CI: 0.02, 0.08).

The results also show that fathers’ parenting involvement and father-child attachment have a significant chain mediating effect on the relationship between maternal positive coparenting consistency and adolescent peer attachment (95% CI: 0.03, 0.05). Maternal positive coparenting consistency was indirectly related to adolescent peer attachment through fathers’ parenting involvement (95% CI: 0.08, 0.13). Maternal positive coparenting consistency was indirectly related to adolescent peer attachment through father-child attachment (95% CI: 0.02, 0.06).

## Discussion

### Analysis

The present study aimed to investigate the relationship between positive coparenting and peer attachment in adolescents and the intermediary role of parental involvement and parent–child attachment based on family systems theory. The results showed that mothers’ positive coparenting positively predicted adolescents’ peer attachment, which was consistent with previous studies (Teubert and Pinquart, 2010; Zou et al., 2020). According to family systems theory, the maternal positive coparenting with the father can promote harmonious coexistence for the whole family, and provide teenagers with security through a harmonious, stable, and reliable family atmosphere, which is an important predictor of adolescents’ peer attachment (Brown and Bakken, 2011; Zemp et al., 2018). When adolescents believe that their parents are stable and reliable, they are more likely to form stable and reliable beliefs towards others, which facilitate healthy interactions with others. Conversely, adolescents may experience interpersonal dilemmas if they believe that others are capricious and unpredictable (Lin et al., 2014; Wang and Cheng, 2014). Therefore, as the main family caregiver, mothers should consider whether their words and deeds are consistent with fathers’ parenting behaviour goals when interacting with teenagers. Parents’ consistent coparenting may provide a warm and harmonious family atmosphere for teenagers, facilitate better family function, and allow them to acquire a more positive interaction style, which will promote healthy adolescent peer attachments.

Second, based on the spillover and crossover effects in family systems theory which indicated that spill over and crossover effects, this study examined the relationships among parenting involvement, mothers’ positive collaborative parenting, and peer attachment. Mothers’ positive collaborative parenting behaviour positively predicted adolescent peer attachment, and predicted adolescent peer attachment through an indirect effect of parenting involvement, which is consistent with previous research findings (Lamb, 2000; Caldera, 2004; Li et al., 2012). The degree of parental involvement determines the extent of social psychological support adolescents receive (Updegraff et al., 2012). Higher levels of support provided adolescents with a sense of security and trust, which were important predictors of adolescent peer attachment (Becker-Stoll et al., 2008). Teenagers will feel more love and care for themselves during this period, allowing them to believe more firmly that they are worthy of being loved and cared for, and that others will interact with them in good faith, which will facilitate positive interactions with others and foster healthy peer attachments (Li et al., 2016).

Third, the results showed that mothers’ positive cooperative parenting behaviour was indirectly related to peer attachment through parent–child attachment during adolescence. As a binary parent–child system, the parent attachment reflects an important parent–child relationship in accordance with family systems theory. Developing peer attachments is influenced by parent–child attachment, an important factor in the internal working model of attachment (Wang et al., 2005; Caldera and Lindsey, 2006), and parent–child attachment can positively affect peer attachment (Doyle et al., 2009). Previous research has also shown that mothers’ positive coparenting can positively affect parent–child attachment (Barnett et al., 2011); when mothers hold a united and consistent attitude towards paternal behaviour, a supportive collaborative parenting situation develops, and teenagers will transfer this coparenting model to their interactions with teachers and peers, resulting in fewer conflicts that can lead to improved interaction styles in adolescents. This could, in turn, lead to improved communication with peers (Brown and Bakken, 2011). Early parent–child attachment relationships are crucial to forming subsequent attachment bonds outside of their family, including peer attachments (Gorrese and Ruggieri, 2012).

Fourth, this study indicated a chain intermediary effect of parental involvement and parent–child attachment on the relationship between maternal positive coparenting and peer attachment, which was consistent with the family systems theory. According to the spill over and crossover effects (Minuchin, 1985; Erel and Burman, 1995), maternal positive coparenting behaviour affects parental involvement, which affects parent–child attachment between adolescents and their parents (Zou et al., 2020). Furthermore, our findings supported the systematic view of family-peer linkages in adolescence (Brown and Bakken, 2011). The positive cooperative relationship between mother and father affect the outcomes of children, especially their friendship.

According to the internal working model of attachment, when adolescents can form a healthy attachment model with their parents, it promotes secure inner attachment that results in teenagers using a healthy model to interact with peers, facilitating healthy peer attachments (Liang and Wang, 2014). Therefore, parents need to pay more attention to mothers’ positive coparenting behaviour. How mothers respond to fathers’ behaviour not only was related to parent involvement and parent–child attachment, which, in turn, was related to adolescent peer attachment. A meta-analysis has shown that parental involvement is correlated with parent–child attachment, and plays an important role in developing parent attachment (De Wolff and Van Ijzendoorn, 1997).

### Limitations and future directions

This section acknowledges several limitations of this study and shows directions for future research. First, this study employed a single self-report method, which should be integrated with interviews, experiments, and others’ evaluation, so as to collect more objective and comprehensive information. Second, this study investigated families with good structure. Future research should examine more family types, especially left behind youth families in the process of urbanization, as a special family form. Moreover, the difference between one child family and more larger families should be given increasing amount of attention in future. Third, this study examined the effect of mothers’ coparenting with fathers. Future studies could consider the role of fathers’ coparenting with mothers, and then focus on how to promote parental positive coparenting behaviour through family education or counseling intervention to promote good communication and healthy growth of teenagers.

### Research implications

Coparenting, involvement, and attachment are important for adolescent development, and consistent parenting behaviours affect parent–child attachment (Neppl et al., 2019). Moreover, previous researchers have found that the parental attachment construct plays a crucial role in peer attachment development (Armsden and Greenberg, 1987; Murphy et al., 2017). Parents’ involvement affects adolescents’ friendship quality and social competence with peers (Updegraff et al., 2001; Ladd and Pettit, 2002). Based on the family system theory, this study innovatively constructed a model including father, mother and adolescents to investigate relations among maternal positive coparenting, parent involvement, parent–child attachment, and peer attachment, comprehensively investigating the influence of mothers’ active coparenting on adolescents, and exploring the ternary system effect of mothers’ active collaborative parenting on the dual paternal and maternal system. Theoretically, the present study extends the understanding of a family–peer system linkage and lays a foundation for future research on how parental positive co-parenting affects parenting involvement and parent–child and peer attachment. Practically, it expands knowledge of parenting behaviour, parent–child relationships, and peer relationships, and has substantial practical significance to family education and family counseling practice.

## Conclusion

This study contributes to our understanding of the chain mediating processes in the association between maternal positive coparenting behaviour and adolescent peer attachment. Based on this exploratory approach, this study examined a mediation model emphasizing the role of mothers’ involvement and parent–child attachment and found that parental involvement and parent–child attachment play a significant mediating role on the associations between mothers’ positive coparenting behaviour and peer attachment through three specific paths: an independent mediating role of parental involvement, an independent mediating role of parent–child attachment, and a chain mediating role of parental investment and parent–child attachment.

## Data availability statement

The original contributions presented in the study are included in the article/Supplementary material, further inquiries can be directed to the corresponding author.

## Ethics statement

The studies involving human participants were reviewed and approved by The Research Ethics Committee of the Institute of Psychology and Behavior, Henan University. Written informed consent to participate in this study was provided by the participants’ legal guardian/next of kin.

## Author contributions

WJ conceptualization, methodology, formal analysis, and writing—review and editing. YY data curation, and writing—original draft preparation. YH validation and investigation. XB supervision and formal analysis. YZ project administration. JL visualization and editing. All authors contributed to the article and approved the submitted version.

## Funding

This study was supported by the Philosophy and Social Science Planning Project in Henan Province, China (Grant Number 2022BSH017).

## Conflict of interest

The authors declare that the research was conducted in the absence of any commercial or financial relationships that could be construed as a potential conflict of interest.

## Publisher’s note

All claims expressed in this article are solely those of the authors and do not necessarily represent those of their affiliated organizations, or those of the publisher, the editors and the reviewers. Any product that may be evaluated in this article, or claim that may be made by its manufacturer, is not guaranteed or endorsed by the publisher.
